# The Mutualistic Side of *Wolbachia*–Isopod Interactions: *Wolbachia* Mediated Protection Against Pathogenic Intracellular Bacteria

**DOI:** 10.3389/fmicb.2015.01388

**Published:** 2015-12-16

**Authors:** Christine Braquart-Varnier, Mine Altinli, Romain Pigeault, Frédéric D. Chevalier, Pierre Grève, Didier Bouchon, Mathieu Sicard

**Affiliations:** ^1^Laboratoire Écologie et Biologie des Interactions – Equipe Écologie, Évolution, Symbiose – UMR CNRS 7267, Université de PoitiersPoitiers, France; ^2^Institut des Sciences de l’Évolution, CNRS—Université de Montpellier-IRD (UMR 5554)Montpellier, France; ^3^IRD 224-Université de Montpellier, Maladies Infectieuses et Vecteurs: Écologie, Génétique, Évolution et Contrôle, Équipe Interaction Parasitaires et AdaptationMontpellier, France; ^4^Texas Biomedical Research Institute, San AntonioTX, USA

**Keywords:** *Wolbachia*, infection, immunocompetence, *Listeria*, Salmonella, terrestrial isopods, *Armadillidium vulgare*, *Porcellio dilatatus*

## Abstract

*Wolbachia* is a vertically transmitted endosymbiont whose radiative success is mainly related to various host reproductive manipulations that led to consider this symbiont as a conflictual reproductive parasite. However, lately, some *Wolbachia* have been shown to act as beneficial symbionts by protecting hosts against a broad range of parasites. Still, this protection has been mostly demonstrated in artificial *Wolbachia*-host associations between partners that did not co-evolved together. Here, we tested in two terrestrial isopod species *Armadillidium vulgare* and *Porcellio dilatatus* whether resident *Wolbachia* (native or non-native) could confer protection during infections with *Listeria ivanovii* and *Salmonella typhimurium* and also during a transinfection with a *Wolbachia* strain that kills the recipient host (i.e., *w*VulC in *P. dilatatus*). Survival analyses showed that (i) *A. vulgare* lines hosting their native *Wolbachia* (*w*VulC) always exhibited higher survival than asymbiotic ones when infected with pathogenic bacteria (ii) *P. dilatatus* lines hosting their native *w*Dil *Wolbachia* strain survived the *S. typhimurium* infection better, while lines hosting non-native *w*Con *Wolbachia* strain survived the *L. ivanovii* and also the transinfection with *w*VulC from *A. vulgare* better. By studying *L. ivanovii* and *S. typhimurium* loads in the hemolymph of the different host-*Wolbachia* systems, we showed that (i) the difference in survival between lines after *L. ivanovii* infections were not linked to the difference between their pathogenic bacterial loads, and (ii) the difference in survival after *S. typhimurium* infections corresponds to lower loads of pathogenic bacteria. Overall, our results demonstrate a beneficial effect of *Wolbachia* on survival of terrestrial isopods when infected with pathogenic intracellular bacteria. This protective effect may rely on different mechanisms depending on the resident symbiont and the invasive bacteria interacting together within the hosts.

## Introduction

Symbioses, defined as intimate interactions between two or more species, can range from mutualism, where both partners benefit from the relationship, to parasitism where one of the partners exploits the other. In order to be maintained and become widespread in the host population, vertically transmitted symbionts adopt some strategies to manipulate their host’s reproduction and/or to provide their host with fitness benefits (Haine, 2008). The advantages of beneficial symbiotic relations can generally be categorized into two main functions: nutrition or protection (Douglas, 2011). In the case of vertically transmitted primary symbionts (i.e., via oocyte), this benefit is usually nutritional, through which the symbiont improves the specialized diet of the host, providing essential nutrients such as amino acids or vitamins (Gross et al., 2009). There is also clear evidence that symbionts can be involved in protection against predators and pathogens (Scarborough et al., 2005; Hedges and Johnson, 2008; Jaenike et al., 2010; Xie et al., 2010). This protection might be achieved with a direct interference with the pathogens or predators by the production of toxic compounds (Davidson et al., 2001; Jaenike and Perlman, 2002). Alternatively this protection can be caused indirectly by the competition between the symbiont and the pathogens for limited resources (Caragata et al., 2013) or by the modulation of host physiology including immune system (de Souza et al., 2009; Kaiser et al., 2010; Weiss et al., 2011). Although the underlying mechanisms of protective symbiosis are not yet unraveled (Douglas, 2011), the existence of protection against pathogens mediated by the presence of a vertically transmitted symbiont is supported by many studies (Haine, 2008; Brownlie and Johnson, 2009).

Even though symbiotic relationships are abundant in both vertebrates and invertebrates, an outstanding diversity of vertically transmitted symbionts has been shown only in the latter (Yen and Barr, 1971; Hurst et al., 1999; Stouthamer et al., 1999; Dunn and Smith, 2001; von der Schulenburg et al., 2001). Being one of the most common vertically transmitted symbiotic bacteria among Cuticulata, *Wolbachia* are found widespread in arthropods and may infect up to 50% of the arthropod species (Weinert et al., 2015). The success of their vertical transmission mainly relies on the manipulation of the reproduction of their hosts in different ways. For example, feminization induced by *Wolbachia* forces infected genetic males to develop in functional females, thus becoming able to transmit *Wolbachia*, resulting in a female bias in the population (Bouchon et al., 2008). Another example of a host reproduction manipulation strategy of *Wolbachia* is the cytoplasmic incompatibility (CI) that gives a fitness advantage to infected females by causing mortality of the embryos when uninfected females copulate with infected males (Serbus et al., 2008). In addition to the ability of the *Wolbachia* to manipulate their hosts reproduction, some interactions have been reported to be detrimental on various host life history traits, including body size (Hoffmann and Turelli, 1988), fecundity (Hoffmann et al., 1990; Fleury et al., 2000), survival (Fleury et al., 2000; Tagami et al., 2001), larval competitiveness (Huigens et al., 2004), mating choice (Rigaud and Moreau, 2004) as well as the hosts’ immunity (Fytrou et al., 2006; Braquart-Varnier et al., 2008; Sicard et al., 2010).

Recent studies showed that *Wolbachia* is not always conflictual and may also act as a mutualist with its hosts by being protective (i.e., increasing their host’s survival during a pathogenic challenge; Teixeira et al., 2008; Gross et al., 2009; Bian et al., 2010; Glaser and Meola, 2010; Zélé et al., 2012; Eleftherianos et al., 2013) or improving nutrition (Hosokawa et al., 2010). The first protective mutualistic effect of the presence of a native *Wolbachia* (*w*Mel) against several viruses has been demonstrated in *Drosophila melanogaster* (Hedges et al., 2008; Teixeira et al., 2008). However, this protection seems limited to viruses (Rottschaefer and Lazzaro, 2012; Ye et al., 2013). On the other hand, in mosquitoes, in addition to the reports of a protective effect against viruses (i.e., Dengue, Chikugunya, in *Aedes aegypti* and *Aedes albopictus*; Moreira et al., 2009), protection against protozoans (Moreira et al., 2009), filarial nematodes (Kambris et al., 2009), as well as two bacteria species (i.e., *Erwinia carotovora* and *Salmonella typhimurium*; Kambris et al., 2009; Ye et al., 2013) have been linked to the presence of *Wolbachia*. However, all these observations were made using mosquitoes artificially transinfected with *w*Mel and *w*Melpop from *Drosophila* (but see Zélé et al., 2012). Though interesting, these situations do not represent natural symbiotic systems where *Wolbachia* would have evolved toward being mutualistic by conferring protection to their host against pathogens.

The *Wolbachia*–isopod symbiotic systems constitute tractable experimental models for transinfection experiments and therefore to study the effects of native and non-native *Wolbachia* on the phenotype of their hosts (Moret et al., 2001; Le Clec’h et al., 2013). Besides, previous studies have been conducted to understand the effects of the *Wolbachia* on the hosts’ immune parameters. These studies have pointed out an immunodepressing effect of the presence of *Wolbachia* during ageing of the terrestrial isopod *Armadillidium vulgare*: 2-years-old females exhibited lower phenoloxidase (PO) activity (Sicard et al., 2010), lower hemocyte density and sometimes even bacteremia in their hemolymph (Braquart-Varnier et al., 2008). The reported presence, in some individuals, of bacteria from the environment suggests that those micro-organisms may constitute a threat in some cases, especially in older animals and/or when the isopod’s immune parameters are low (Braquart-Varnier et al., 2008). However, in younger individuals (i.e., 1-year-old animals) of two isopod species (*A. vulgare* and *Porcellio dilatatus*), no such negative effects of *Wolbachia* on immune parameters were observed suggesting that *Wolbachia* would only cause immunodepression in older animals (Sicard et al., 2010; Pigeault et al., 2014).

Considering the contrasted influence of *Wolbachia* on isopods’ (i) immune parameters (i.e., PO activity, hemocyte density, phagocytosis, proportion of hemocyte types; Braquart-Varnier et al., 2008; Sicard et al., 2010; Pigeault et al., 2014) and (ii) immune gene expression (Chevalier et al., 2012), the impact of the presence of *Wolbachia* on their host immunocompetence when facing a pathogen had to be directly assessed. As some environmental bacteria were detected, in some cases, in the isopod hemolymph, it appeared pertinent to study the impact of resident *Wolbachia* on the survival of terrestrial isopods against bacterial pathogens by injecting them directly into the hemolymph. Despite comprehensive investigations, no specific cultivable bacterial pathogens of terrestrial isopod have been described to date (Braquart-Varnier and Sicard, personal observation). Thus, we chose to investigate this question with two intracellular bacterial pathogens ecologically relevant and already demonstrated to be pathogenic toward arthropods: the Gram-positive *Listeria ivanovii* and the Gram-negative *S. typhimurium*, which were both detected in soil samples (Jacobsen and Bech, 2012; Sauders et al., 2012). In parallel with our investigation on ‘conventional’ bacterial pathogens, we also studied the impact of a resident vertically transmitted *Wolbachia* on the detrimental effect caused by the transinfection of the *w*VulC strain from *A. vulgare* to *P. dilatatus* (Le Clec’h et al., 2012). Indeed such transinfection has led to the death of all asymbiotic individuals (i.e., without *Wolbachia*; Le Clec’h et al., 2012). Resident *Wolbachia* (either native or non-native) might affect the host survival in case of such multiple infections (i.e., the resident *Wolbachia* strain and the invasive strain) in two main ways: (i) causing a decrease in *w*VulC induced mortality due to negative interference between the two *Wolbachia* strains, (ii) or causing an increase in *w*VulC induced mortality due to the additive costs that can result from multiple infections (López-Villavicencio et al., 2011). All the three “pathogens” used in this study are considered to share a similar intracellular niche with the resident *Wolbachia* and thus can potentially compete with the resident symbiont for resources (Caragata et al., 2013; Sicard et al., 2014b). Such competition could in return decrease the success of the pathogens (Loker et al., 2004). *Wolbachia* could also interfere with pathogenic bacteria directly via the production of toxic compounds or in a more indirect way, by modulating the host physiology (Kaiser et al., 2010), particularly the host immune system (Kambris et al., 2009).

The present study aimed to test whether resident *Wolbachia* (either native or not) can modulate the survival of the terrestrial isopods when infected with the pathogenic intracellular bacteria *L. ivanovii* and *S. typhimurium* as well as after the transinfection with *Wolbachia* (*w*VulC). We showed in both *A. vulgare* and *P. dilatatus* that the presence of a native *Wolbachia* can increase their ability to survive pathogenic bacterial infections. We thus reveal a ‘mutualistic side’ of *Wolbachia*–isopod interactions, which were considered until now as quite conflictual ones.

## Materials and Methods

### Biological Material

#### Isopod Lines

We studied the impact of resident *Wolbachia* on their host survival when infected with intracellular pathogenic bacteria with lines of two terrestrial isopod host species. These two host species were chosen because each of them allowed to test the effect of different parameters: (i) the model *A. vulgare* allowed us to test the impact of the feminizing *Wolbachia w*VulC but also of their different host’s population of origin while (ii) the model *P. dilatatus* allowed us to test the effect of two CI-inducing *Wolbachia* genotypes (native versus non-native) on the same host genetic background. In *A. vulgare* lines, the impact of *Wolbachia* on the survival when injected with the pathogenic bacteria could not be tested in males as no males of this species were infected with *Wolbachia* due to the feminization process. However, for *P. dilatatus*, it was possible to test the effect of gender as they host CI-inducing strains, *w*Dil and *w*Con, which infect both sexes (Sicard et al., 2014a).

In a previous study, Le Clec’h et al. (2013), had introduced by injection the native *Wolbachia* strain (*w*Dil) isolated from a *P. dilatatus* symbiotic line (Sicard et al., 2014a), and a non-native CI *Wolbachia* strain (*w*Con) isolated from *Cylisticus convexus* (Moret et al., 2001) in recipient individuals from the asymbiotic *P. dilatatus* line (dilatatus A line; **Table 1**; Sicard et al., 2014a). Both *Wolbachia* are since then stably transmitted from injected mothers to further generations resulting in two independent lines maintained in the laboratory so called ‘dilatatus A-*w*Con’ and ‘dilatatus A-*w*Dil’ (**Table 1**).

**Table 1:** Isopod lines used in this study and their main characteristics: gender of studied animals, their origins, their symbiotic condition [asymbiotic, symbiotic (naturally or experimentally infected)], the *Wolbachia* strain that they harbor.

Species	Line	Gender	Origin	*Wolbachia* status	Resident *Wolbachia* strain	Pathogen injections
*Armadillidium vulgare*	WXa	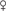	Denmark	Asymbiotic	—	*Salmonella typhimurium* and *Listeria ivanovii*
	WX*w*	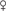	Denmark	Symbiotic	*w*VulC^WX^	*S. typhimurium* and *L. ivanovii*
	BF	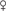	France	Asymbiotic	—	*S. typhimurium* and *L. ivanovii*
	BF*w*VulC	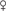	BF line	Experimentally infected	*w*VulC^ZN^	*S. typhimurium* and *L. ivanovii*
	ZN	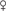	France	Symbiotic	*w*VulC^ZN^	*S. typhimurium* and *L. ivanovii*
*Porcellio dilatatus*	*dilatatus* A	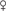 and 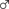	France	Asymbiotic	—	*w*VulC^WX^, *S. typhimurium* and *L. ivanovii*
	*dilatatus* A- *w*Dil	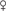 and 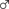	*dilatatus* A line	Experimentally infected	*w*Dil	*w*VulC^WX^, *S. typhimurium* and *L. ivanovii*
	*dilatatus* A- *w*Con	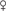 and 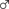	*dilatatus* A line	Experimentally infected	*w*Con	*w*VulC^WX^, *S. typhimurium* and *L. ivanovii*

We used two *A. vulgare* asymbiotic lines (WXa from Helsingor and BF from Nice; **Table 1**) and two *A. vulgare* symbiotic lines infected with *w*VulC (ZN from Celles-Sur-Belles and WX*w* from Helsingor; **Table 1**). We also created the BF*w*VulC line to have BF individuals infected with *Wolbachia w*VulC with the same genetic background as BF asymbiotic population. This line was generated by injection of *Wolbachia* from ZN females in BF recipient females following the procedure described in Le Clec’h et al. (2013). Recipient females were then crossed with BF males to create this new line. Then, females and males from the BF*w*VulC line are crossed together at each generation to maintain the line. Animals from the BF*w*VulC line are infected with *Wolbachia w*VulC but have the ‘genetic background’ of BF asymbiotic line. The sex-ratio deviance due to *Wolbachia* infection in BF*w*VulC line was similar to the one observed in ZN line (data not shown). All the individuals from the different lines were grown at 20°C, in plastic breeding boxes, on humid soil and fed with dead lime-tree leaves. All animals used in this study were 1-year-old.

#### Pathogenic Intracellular Bacteria

Two phylogenetically distant intracellular pathogenic bacteria species, *L. ivanovii* (Phylum: Firmicutes) and *S. typhimurium* (Phylum: Proteobacteria), were injected into *A. vulgare* and *P. dilatatus* individuals from the different lines. *L. ivanovii* are Gram-positive intracellular bacteria, which are facultative anaerobe, non-spore forming rods (Vázquez-Boland et al., 2001). *L. ivanovii* has been shown to establish an intracellular infection causing moderate mortality in *D. melanogaster* (Rottschaefer and Lazzaro, 2012). The Gram-negative bacteria from the serotype *Salmonella enterica* serotype Typhimurium are facultative aerobe non-spore forming bacilli (Velge et al., 2012). *S. typhimurium* is considered as having a broad host range since they are able to infect the cells of many vertebrates (Velge et al., 2012) as well as invertebrates, such as *D. melanogaster* (Rottschaefer and Lazzaro, 2012). The strain used in this study expressed constitutively GFP.

### *Salmonella* and *Listeria* Injections

#### Bacterial Cultures

For *S. typhimurium* and *L. ivanovii* injections, bacteria from glycerol stocks were cultured overnight at 37°C in liquid LB medium (Luria Bertani Broth Base, Invitrogen) or in BHI medium (Brain-Heart Infusion, BD) respectively. These cultures were then used to grow bacterial colonies on LB plates or BHI plates at 37°C (LB or BHI with 15 g/L agar) until colonies reached a 5 mm diameter. Before each injection experiment, one colony of either *S. typhimurium* or *L. ivanovii* from solid cultures was added to 5 mL of LB or BHI liquid medium and incubated overnight at 37°C. One hundred microliter of the overnight culture was added to 5 mL of LB or BHI and incubated at 37°C to reach a 0.7 optical density (OD) at 600 nm. One milliliter of the 0.7 OD culture was then centrifuged (at 13000 g, 4°C, 2 min). The supernatant was disposed and the pellet was resuspended in 1 mL of fresh LB or BHI medium. The resulting suspension contained around 10^5^ bacteria/μL for *S. typhimurium* and 10^6^ bacteria/μL for *L. ivanovii*. The *S. typhimurium* suspension was then diluted by 10 in order to obtain a 10^4^ bacteria/μL suspension used for challenging both the *A. vulgare* and *P. dilatatus* individuals. Regarding the *L. ivanovii*, the initial 10^6^ bacteria/μL suspension was used to challenge the *A. vulgare* individuals. Due to the higher susceptibility of *P. dilatatus* to *L. ivanovii* (data not shown), the *L. ivanovii* suspension was diluted by 10 to reach 10^5^ bacteria/μL to challenge *P. dilatatus*.

For each independent replicate (for both survival and pathogen multiplication experiments), all the bacterial cultures were prepared separately. Additionally, to check the actual number of injected bacteria, serial dilutions were made to obtain around 1 bacteria/μL, and 100 μL of this dilution was streaked on solid LB plates or BHI plates depending on the bacteria.

#### Survival Assays

A Hamilton syringe with fine glass needle was used to inject 1 μL of the bacterial suspension (*S. typhimurium* or *L. ivanovii*) or 1 μL of the control solution (LB or BHI sterile liquid medium respectively) into the general cavity. All batches comprised six individuals of each line (WXa, WX*w*, BF, BF*w*VulC, ZN, dilatatus A, dilatatus A-*w*Con, and dilatatus A-*w*Dil) and were independently replicated five times (*n* = 30). For each replicate, three individuals from each line received the control treatment (*n* = 15 per line). After the injections of *L. ivanovii* or *S. typhimurium*, the animals from the same replicate were kept in a plastic box with moist paper and checked every 4 h to record the mortality during 144 h for *S. typhimurium* challenged individuals or 200 h for *L. ivanovii* challenged individuals (as the mortality appeared later for *L. ivanovii* injected isopods).

#### Pathogen Multiplication in Asymbiotic versus Symbiotic Animals

In order to compare the *L. ivanovii* and *S. typhimurium* loads between symbiotic (BF*w*VulC, dilatatus A-*w*Dil) and asymbiotic (BF, dilatatus A) lines, animals were injected in three independent replicates with *S. typhimurium* (BF*w*VulC *n* = 20, BF *n* = 20; dilatatus A *n* = 32, dilatatus A-*w*Dil = 24) or with *L. ivanovii* (BF*w*VulC *n* = 30; BF *n* = 30; dilatatus A *n* = 15; dilatatus A-*w*Dil *n* = 15). Five microliters of hemolymph of each injected animals were sampled 24 h post-injection (PI). Sampled hemolymph from *S. typhimurium* infected isopods was diluted by 10^6^ in LB to reach a countable number of *S. typhimurium* colonies and 100 μL of these dilutions were streaked on two independent LB plates. All colonies grown on the plates were morphologically similar. However, to check whether the colonies on the plates were actually *S. typhimurium*, we spread them on a slide and checked under epifluorescence microscope (Olympus IX81) at λ = 411 nm for GFP expression. Sampled hemolymph from *L. ivanovii* infected isopods were added to 95 μL of BHI and directly streaked on BHI plates (no further dilution needed due to the low amount of *L. ivanovii* in hemolymph). The plates were then incubated overnight and the CFUs were counted the next day. To check whether the colonies showing the proper morphology all belonged to *L. ivanovii*, we sequenced randomly some of them on 16S rDNA.

### Transinfection of *P. dilatatus* with *w*VulC from *A. vulgare*: Consequences on Survival and Mobility

Asymbiotic dilatatus A along with symbiotic dilatatus A-*w*Con and dilatatus A-*w*Dil males (**Table 1**) were injected either with the *w*VulC suspension (obtained from crushed ovaries of infected females) or with the control suspension (obtained from crushed ovaries of uninfected females). To do so, a Hamilton syringe with a fine glass needle was used to inject 2 μL of filtered ovaries suspension from WX*w* or WXa females (**Table 1**) prepared as described in Le Clec’h et al. (2013) into the general cavity of the animals, through a small hole pierced at the posterior part of the animal. For each treatment, three independent replicates were conducted (*n* = 21 received *w*VulC injection for dilatatus A, dilatatus A-*w*Con, and dilatatus A-*w*Dil while *n* = 27 received the control treatment).

After injection of *w*VulC into dilatatus A, dilatatus A-*w*Con, and dilatatus A-*w*Dil individuals, injected animals from each replicate were kept at 20°C in a plastic rearing box on humid soil and fed with dried lime-tree leaves soil. The survival was recorded every 15 days starting at day 1 until day 112. In addition, a mobility test was performed on the survivors every 15 days, starting from day 60 until the day 105, by measuring the time the animals move in a Petri dish during a period of 180 s (Le Clec’h et al., 2012).

#### Detection and Density of *Wolbachia*

We quantified the density of *Wolbachia* by qPCR, both in the suspensions injected to *P. dilatatus* (i.e., when *w*VulC is injected as the invasive pathogen) and in the animals that received pathogenic bacterial injections (i.e., quantification of the resident *Wolbachia*: *w*Con, *w*Dil in *P. dilatatus* and *w*VulC in *A. vulgare*) using either ovary or leg samples. The latter can be sampled without killing the animals allowing us to use the same animal both to measure the quantity of *Wolbachia* and to inject pathogens. Total DNA was extracted using the protocol described by Kocher et al. (1989) and the Nanodrop 1000 spectrophotometer was used to estimate the total DNA concentration and quality (ratios OD 260/280 nm). Reactions of qPCR were performed, as previously described in Le Clec’h et al. (2012), using Roche LightCycler 480 to measure the copy number of the *Wolbachia* surface protein (*wsp*) gene. In short, each 10 μL reaction contained 5 μL of SYBRGreen Master Mix (Roche), 0.5 μL of 10 μM specific primers *wsp*208f (5′-TGG-TGC-AGC-ATT-TAC-TCCAG-3′) and *wsp*413r (5′-TCG-CTT-GATAAG-CAA-AAC-CA-3′), 3 μL of sterile water and 1 μL of DNA (corresponding to a range of 5–50 ng). The thermal cycle starts with a 10 min initial denaturation at 95°C followed by 45 cycles of 10 s of denaturation at 95°C, 10 s of annealing at 60°C and 20 s of elongation at 72°C. The specificity of the PCR product was verified with a melting curve (65–97°C) that was recorded at the end of the each reaction. The *wsp* copy number was estimated with the help of the standard curve plotted using a dilution of the *wsp* purified PCR product (2.63 × 10^3^ *wsp*.copies μL^-1^). The *wsp* copy number was then divided by the total DNA amount of the sample in ng in order to obtain normalized values for comparison between samples. For each sample two independent technical replicates were made.

### Statistical Analyses

R 3.2.2 was used for all of the statistical analysis.

#### *Wolbachia* Titer

Shapiro-Wilk and Levene’s tests were conducted to check normality and homoscedasticity of the number of *wsp* copy/ng of total DNA. A *t*-test with Bonferroni correction for multiple testing was used when the data followed a normal distribution and variances of the samples were homogenous. A Wilcoxon-rank test was performed when the data was not normally distributed.

#### Isopod Survival After *L. ivanovii* and *S. typhimurium* Injections

A global mixed effects Cox proportional hazards model was fitted using the “coxme” R package. Bacterial injection (i.e., control/*Salmonella* or *Listeria*), *Wolbachia* status (presence/absence for *A. vulgare*; presence of different *Wolbachia* strains/absence of *Wolbachia* for *P. dilatatus*) as well as gender (for *P. dilatatus*) and population of origin (for *A. vulgare*) were included in the models as fixed effects. As the experiments were performed on different independent groups of individuals, a block effect was included as a random effect in the models. The latter analysis showed a low variance between independent groups (i.e., blocks) indicating the repeatability of the experiments. Each model was also fitted without control groups. This allowed us to compare the survival of the different asymbiotic and symbiotic lines when infected with the pathogenic bacteria. Survival of the different lines was compared pairwise to assess the difference between their survival times using the log-rank test.

#### Consequences of *w*VulC Transinfection on *P. dilatatus* Life History Traits

Models were fitted for the days 60, 75, and 105 PI in order to test differences in survival during the course of the infection of *P. dilatatus* by *w*VulC. Survival was modeled using a mixed effects Cox proportional hazards model as described above. For the analysis of the mobility, the ‘nlme’ package (version 3.1-120) was used to fit a mixed effect linear model with a random block effect. In both analyses, first a global model including all of the three lines (i.e., dilatatus A, dilatatus A *w*Con, and dilatatus A *w*Dil) was performed. Then, further analysis was made to clarify the effect of the presence of each of the *Wolbachia* strains (i.e., *w*Dil, *w*Con) on mobility and survival, when the host was infected with *w*VulC. Additionally, the log-rank test was used to compare the survival times of each lineage pairwise.

## Results

### *Wolbachia* Density in Symbiotic *A. vulgare* and *P. dilatatus*

**FIGURE 1:**
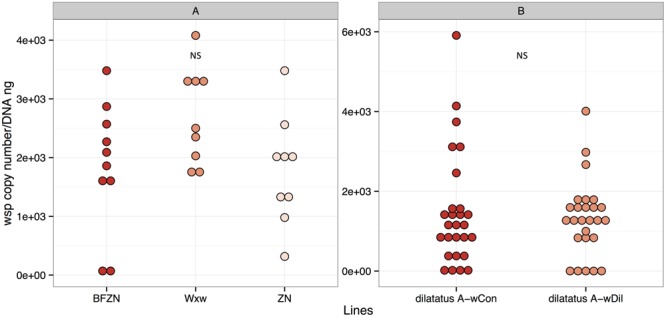
***Wolbachia* density in *Armadillidium vulgare***(A)** and *Porcellio dilatatus***(B)**.** The qPCR quantifications of *wsp* copy number/DNA ng in BF*w*VulC, ZN and WX*w* lineages and dilatatus A- *w*Con, dilatatus A- *w*Dil lineages were made, using leg and ovary samples respectively. NS, not significant.

Females of symbiotic *A. vulgare* lines were found infected with *Wolbachia* as expected (using leg samples; **Figure 1A**). The *Wolbachia* density did not differ significantly between the lines that are naturally (ZN, WX*w*) or experimentally (BF*w*VulC) infected with *w*VulC (*t*-test with Bonferroni correction; ZN and BF*w*VulC: *t* = 0.133, df = 17, *p* = 1.00; WX*w* and BF*w*VulC: *t* = 1.903, df = 17, *p* = 0.19; ZN and WX*w*: *t* = 2.228, df = 16, *p* = 0.16; **Figure 1A**).

The symbiotic *P. dilatatus* individuals from the lines dilatatus A-*w*Con and dilatatus A-*w*Dil were also proved to be all infected with *Wolbachia* using ovary samples (**Figure 1B**). The density of *Wolbachia* in both lines was similar as already reported in Pigeault et al. (2014) (Wilcoxon-rank sum test, *W* = 361, *p* = 0.866).

The number of *wsp* copies in the *w*VulC bacterial suspensions injected for transinfection of *P. dilatatus* was around of 1 × 10^6^ *wsp* copy/μL.

### The Impact of *Wolbachia* on the Survival of Terrestrial Isopods Infected with *L. ivanovii* and *S. typhimurium*

#### Global Survival Analysis

For both isopod species, the global survival models including all treatment groups (controls without injection of pathogens included), showed that *L. ivanovii* and *S. typhimurium* injections always significantly reduced the survival of terrestrial isopods (**Table 2**): both *L. ivanovii* and *S. typhimurium* were pathogenic for both isopod host species. The presence or absence of pathogenic bacteria in the injection explained an important part of the deviance between treatments. However, this was not the only significant explanatory factor. In the global model indeed, the symbiotic status (i.e., individual infected or not with *Wolbachia*) of the host also explained an important part of the deviance, with the exception of *P. dilatatus* individuals injected with *L. ivanovii* for which *Wolbachia* presence did not influence the survival (**Table 2**).

**Table 2:** Survival analyses of the different *A. vulgare* and *P. dilatatus* lines when injected with *S. typhimurium* or *L. ivanovii*.

	*S. typhimurium injections*			*L. ivanovii injections*		
	df	Deviance	*p*	df	Deviance	*p*
***P. dilatatus***						
With control						
Bacterial injection	1	77.193	**<0.001**	1	35.156	**<0.001**
*Wolbachia* status	2	22.244	**<0.001**	2	0.877	0.645
Gender	1	6.191	**<0.001**	1	7.863	**0.005**
Without control						
*Wolbachia* status	2	22.244	**<0.001**	2	0.739	0.691
Gender	1	6.191	**0.013**	1	10.396	**0.001**
***A. vulgare***						
With control						
Bacterial injection	1	20.141	**<0.001**	1	6.322	**0.012**
*Wolbachia* status	1	5.737	**0.017**	1	24.630	**<0.001**
Population of origin	2	6.860	**0.032**	2	10.606	**0.005**
Without control						
*Wolbachia* status	1	7.943	**0.001**	1	15.302	**<0.001**
Population of origin	2	7.568	**0.023**	2	31.630	**<0.001**

*Isopods were injected either with *S. typhimurium* or *L. ivanovii* and their survival was recorded. A mixed effect Cox proportional hazards model was fitted with and without the controls using the survival data of the lineages to estimate the effects of the treatment, the presence of *Wolbachia*, the different *Wolbachia* strains, the gender (only in *P. dilatatus*), the population of origin (only in *A. vulgare*) on the survival and the replicate blocks as a random effect. In bold, statistically significant values (*p* < 0.05).*

**FIGURE 2:**
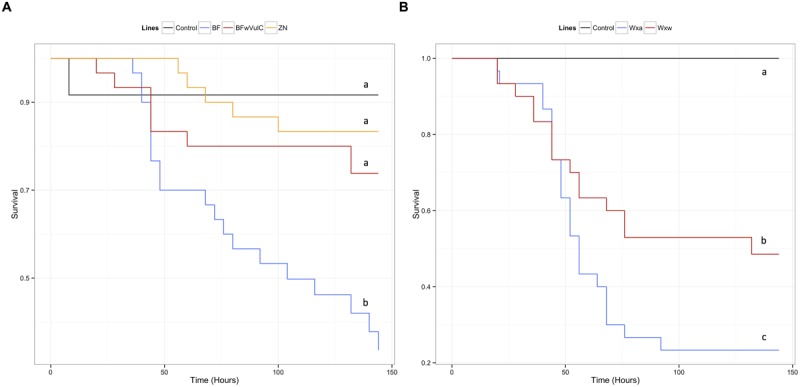
**Survival of the different *A. vulgare* lines when infected with *S. typhimurium*.** A Cox proportional hazards model has been fitted using the survival data compared symbiotic and asymbiotic survival **(A)** BF*w*VulC and ZN/BF and **(B)** WX*w*/WXa *A. vulgare* lines after being injected with pathogenic bacteria *S. typhimurium*. Control groups were injected with liquid medium (LB). Different letters indicate significant differences between the survival curves of different lineages based on log-rank test *p* < 0.05.

**FIGURE 3:**
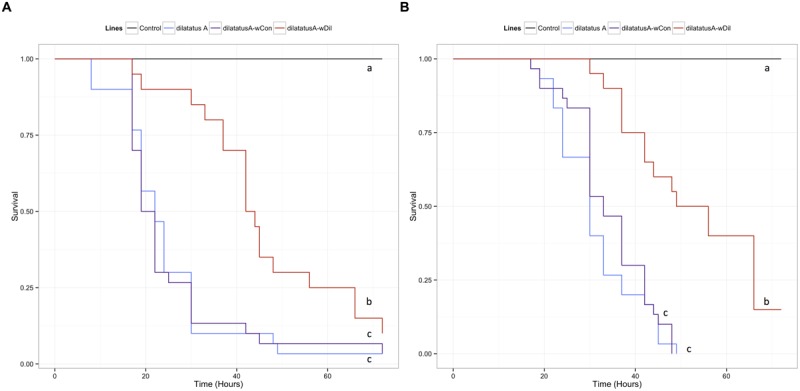
**Survival of the different *P. dilatatus* lines when infected with *S. typhimurium*.** A Cox proportional hazards model was fitted using the survival data of symbiotic (*dilatatus* A-*w*Con, dilatatus A-*w*Dil) and asymbiotic (*dilatatus* A) *P. dilatatus* lines after being injected with pathogenic bacteria *S. typhimurium*. Control groups were injected with liquid medium (LB). Results for females **(A)** and for males **(B)** are presented separately. Different letters indicate significant differences between the survival curves of different lineages based on log-rank test *p* < 0.05.

Other explanatory factors were specific to each of the isopod species. In *A. vulgare*, the effect of the population of origin showed that this factor also significantly explained a part of the deviance (**Table 2**; **Figures 2** and **5**). In *P. dilatatus*, the global model showed that the survival depended significantly on the gender when injected with *L. ivanovii* or *S. typhimurium*: females survived better than males (**Table 2**; **Figures 3** and **6**).

### Impact of *Wolbachia* on *S. typhimurium* Infections

#### Survival

A reduced submodel was fitted after excluding the control groups (i.e., injection of sterile culture media) to evaluate which parameter (gender, population of origin, *Wolbachia* status) still explained significantly the deviance in the survival of animals infected with *S. typhimurium*.

In *A. vulgare*, the presence of *Wolbachia* explained an important part of deviance in survival: symbiotic lines survived the *S. typhimurium* infection better than asymbiotic ones (**Table 2**). Originating from the same line and only differing in their symbiotic condition, BF*w*VulC individuals survived better than BF ones (log-rank test: χ^2^ = 7.2, df = 1, *p* = 0.007, **Figure 2A**). On the other hand, BF*w*VulC showed a very similar survival pattern to the other symbiotic lineage ZN (log-rank test: χ^2^ = 0.7, df = 1, *p* = 0.405, **Figure 2A**), even though they did not come from the same line but have the same *Wolbachia* strain. Similarly, the symbiotic lineage WX*w* survived better than the asymbiotic WXa originated from the same initial population (log-rank test: χ^2^ = 3.8, df = 1, *p* = 0.049, **Figure 2B**).

In *P. dilatatus*, the reduced submodel showed that both the *Wolbachia* status and at a lower extent the gender, explained significantly a part of the deviance (**Table 2**). The survival of *P. dilatatus* when injected with *S. typhimurium* differed not only between symbiotic and asymbiotic animals, but also depending on the resident *Wolbachia* strain present (i.e., native *w*Dil and non-native *w*Con). Pairwise comparison of survival data with log-rank test showed that dilatatus A-*w*Dil animals infected with native *Wolbachia* survived better than both dilatatus A-*w*Con and asymbiotic dilatatus A ones. This pattern was observed for females and males (for females, dilatatus A and dilatatus A-*w*Dil: log-rank test, χ^2^ = 9.4, df = 1, *p* = 0.002; dilatatus A-*w*Con and dilatatus A-*w*Dil: log-rank test, χ^2^ = 7.7, df = 1, *p* = 0.006, **Figure 3A**; for males, dilatatus A and dilatatus A-*w*Dil: log-rank test, χ^2^ = 25.3, df = 1, *p* < 0.001, dilatatus A-*w*Con and dilatatus A-*w*Dil: log-rank test, χ^2^ = 22.1, df = 1, *p* < 0.001, **Figure 3B**). However, the survival of asymbiotic dilatatus A and symbiotic dilatatus A-*w*Con was not significantly different (females: dilatatus A and dilatatus A-*w*Con: log-rank test, χ^2^ = 0.2, df = 1, *p* = 0.683, **Figure 3A**; males: dilatatus A and dilatatus A-*w*Con: log-rank test, χ^2^ = 0.6, df = 1, *p* = 0.435, **Figure 3B**). These results showed that only *P. dilatatus* individuals harboring the native *Wolbachia* strain *w*Dil survived better than the other lines when infected with *S. typhimurium*.

#### Pathogen Load

**FIGURE 4:**
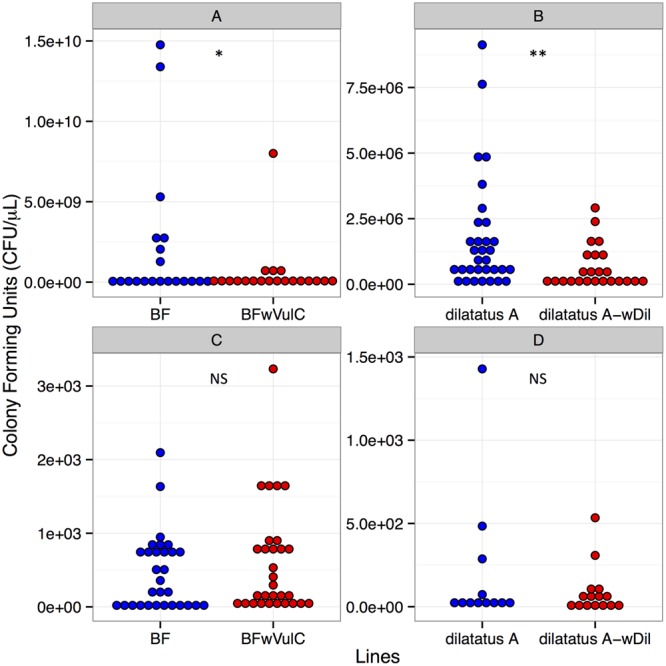
***Salmonella typhimurium***(A,B)** and *Listeria ivanovii***(C,D)** CFUs in *A. vulgare* and *P. dilatatus*.** CFUs of *S. typhimurium* from *A. vulgare* hemolymph **(A)**, from *P. dilatatus* hemolymph **(B)**, CFUs of *L. ivanovii* from *A. vulgare* hemolymph **(C)** and CFUs of *L. ivanovii* from *P. dilatatus* hemolymph **(D)**. ^∗^*p* < 0.05, ^∗∗^*p* < 0.01. NS, not significant.

Twenty-four hours PI, hemolymph samples were taken from animals injected with *S. typhimurium* to compare the pathogen load (i.e., CFUs) between asymbiotic (BF, dilatatus A) and symbiotic (BF*w*VulC, dilatatus A-*w*Con, dilatatus A-*w*Dil) animals. In both *A. vulgare* and *P. dilatatus*, the pathogen load in the hemolymph was higher than the amount that was initially injected (at least 400 times higher). Moreover, in both *A. vulgare* and *P. dilatatus*, CFUs from *S. typhimurium* was significantly higher in asymbiotic animals compared to the symbiotic ones (BF/BF*w*VulC: *W* = 274, *p* = 0.043, **Figure 4A**; dilatatus A/dilatatus A-*w*Dil *W* = 631.5, *p* = 0.006, **Figure 4B**).

### Impact of *Wolbachia* on *L. ivanovii* Infections

#### Survival

**FIGURE 5:**
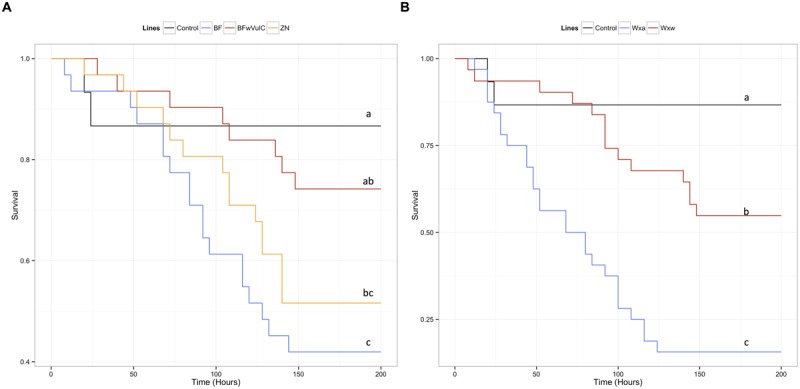
**Survival of the different *A. vulgare* lines when infected with *L. ivanovii*.** A Cox proportional hazards model has been fitted using the survival data compared symbiotic and asymbiotic survival **(A)** BF*w*VulC and ZN/BF and **(B)** WX*w*/WXa. *A. vulgare* lines after being injected with pathogenic bacteria *L. ivanovii*. Control groups were injected with liquid medium (BHI). Different letters indicate significant differences between the survival curves of different lineages based on log-rank test *p* < 0.05.

In *A. vulgare*, similar to the global model, the reduced submodel showed that both the *Wolbachia* status and the origin of the population explained significantly a part of the deviance (**Table 2**). Pairwise comparison of survival data showed that symbiotic BF*w*VulC females survived significantly better than asymbiotic BF ones (log-rank test, χ^2^ = 4.7, df = 1, *p* = 0.029, **Figure 5A**) while survival of BF*w*VulC and ZN did not differ from each other (log-rank test, χ^2^ = 3.2, df = 1, *p* = 0.075, **Figure 5A**). In addition, symbiotic WX*w* females survived significantly better than asymbiotic WXa when injected with *L. ivanovii* (log-rank test, χ^2^ = 16.7, df = 1, *p* < 0,001, **Figure 5B**).

**FIGURE 6:**
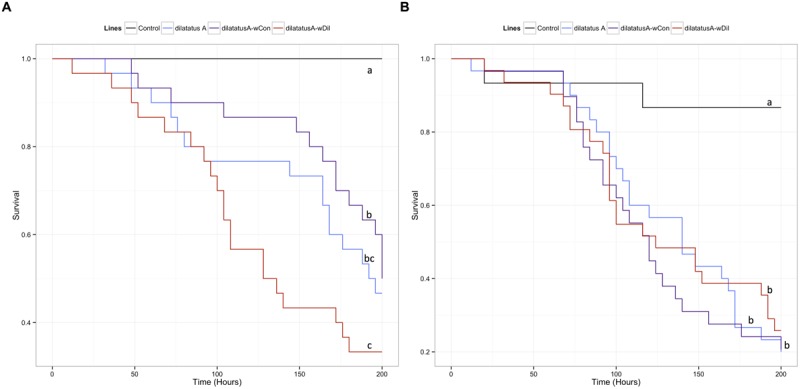
**Survival of the different *P. dilatatus* lines when infected with *L. ivanovii*.** A Cox proportional hazards model was fitted using the survival data of symbiotic (*dilatatus* A-*w*Con, *dilatatus* A-*w*Dil) and asymbiotic (*dilatatus* A) individuals after being injected with pathogenic bacteria *L. ivanovii*. Results for females **(A)** and for males **(B)** are presented separately. Control groups were injected with liquid medium (BHI). Different letters indicate significant differences between the survival curves of different lineages based on log-rank test. *p* < 0.05.

In *P. dilatatus*, a significant part of the deviance in survival was explained in the reduced submodel by the factor ‘gender’: females surviving better than males (**Table 2**). In this case, the *Wolbachia* status did not significantly affect the survival in the model (*p* = 0.691; **Table 2**). However, pairwise comparison of survival data showed a weak effect of *Wolbachia* status; there was actually no significant difference between the survival of asymbiotic and symbiotic females infected with *L. ivanovii* (dilatatus A and dilatatus A-*w*Con: log-rank test, χ^2^ = 0.3, df = 1, *p* = 0.579; dilatatus A and dilatatus A-*w*Dil: log-rank test, χ^2^ = 1.8, df = 1, *p* = 0.180; **Figure 6A**). However, there was a significant difference in survival between females infected with different *Wolbachia*: dilatatus A-*w*Con females survived better than dilatatus A-*w*Dil ones (log-rank test, χ^2^ = 4, df = 1, *p* = 0.046; **Figure 6B**). This pattern was not confirmed in males for which no difference due to the presence of any of the *Wolbachia* strains was detected (log-rank test: χ^2^ = 0.4, df = 2, *p* = 0.813; **Figure 6B**).

#### Pathogen Load

Twenty-four hours PI, hemolymph was sampled from *L. ivanovii* injected animals to compare the pathogen loads between asymbiotic (BF, dilatatus A) and symbiotic (BF*w*VulC, dilatatus A-*w*Con, dilatatus A-*w*Dil) animals. The mean of the pathogen load in the hemolymph was more than 60 times lower than the initially injected *L. ivanovii* amount. No significant difference between asymbiotic and symbiotic animals was detected in any of the isopod models (for *A. vulgare*: *W* = 425.5, *p* = 0.723, **Figure 4C**; for *P. dilatatus*: *W* = 90, *p* = 1, **Figure 4D**).

### Influence of Resident *Wolbachia* on Invasive *Wolbachia*

#### Survival

**FIGURE 7:**
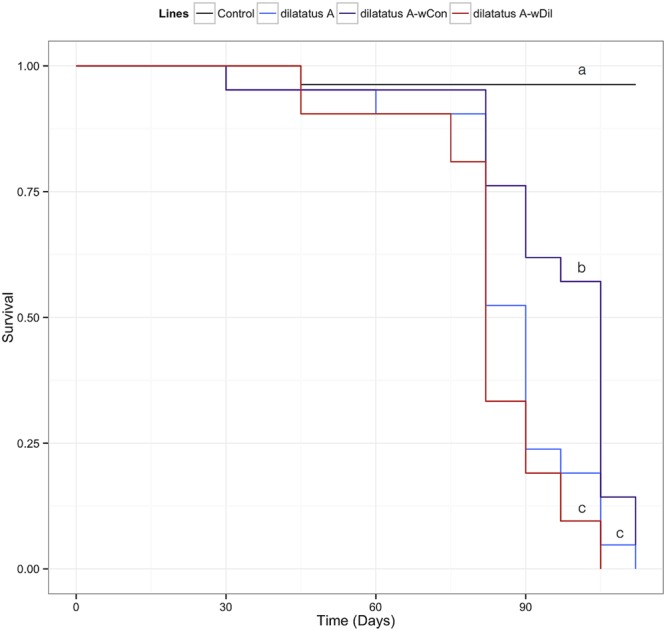
**Influence of resident *Wolbachia* on the survival of *P. dilatatus* infected with *w*VulC.** *Porcellio dilatatus* males were injected with invasive *w*VulC *Wolbachia* strain and their survival was recorded every 15 days. A Cox proportional hazards model was fitted. Different letters indicate significant differences between the survival curves of different lineages based on log-rank test. *p* < 0.05.

The negative effect of the *w*VulC infection on the survival of the animals was detected from day 90 PI (**Figure 7**; **Table 3**). Pairwise comparisons of survival data with log-rank test showed that the differential effect of the *Wolbachia* status (asymbiotic, dilatatus A-*w*Dil, or dilatatus A-*w*Con) became apparent at day 105 PI (**Table 3**): globally, dilatatus A-*w*Con survived significantly longer than dilatatus A individuals and dilatatus A-*w*Dil ones (log-rank test, dilatatus A-*w*Dil and dilatatus A-*w*Con: χ^2^ = 12.2, df = 1, *p* < 0.001; dilatatus A and dilatatus A-*w*Con: χ^2^ = 5.8, df = 1, *p* = 0.015; **Figure 7**).

**Table 3:** Influence of resident *Wolbachia* on the survival and mobility of *P. dilatatus* infected with *w*VulC.

	Day 60 PI			Day 75 PI			Day 105 PI		
	df	Deviance	*p*	df	Deviance	*p*	df	Deviance	*p*
**Comparisons between *dilatatus* A, *dilatatus* A-*w*Con, and *dilatatus* A-*w*Dil**					
Mobility									
Bacterial injection	1	15.245	**<0.001**	1	26.701	**<0.001**	1	18.135	**<0.001**
*Wolbachia* status	2	3.535	0.171	2	6.635	**0.036**	2	6.080	**0.048**
Survival									
Bacterial injection	1	0.604	0.436	1	1.285	0.257	1	42.591	**<0.001**
*Wolbachia* status	2	0.993	0.608	2	1.576	0.448	2	9.218	**0.01**
**Comparisons between *dilatatus* A-*w*Con and *dilatatus* A-*w*Dil**					
Mobility									
Bacterial injection	1	11.681	**0.001**	1	20.181	**<0.001**	1	18.138	**<0.001**
*Wolbachia* status	1	1.612	0.204	1	5.724	**0.017**	1	6.080	**0.014**
Survival									
Bacterial injection	1	0.214	0.643	1	3.413	0.064	1	32.185	**<0.001**
*Wolbachia* status	1	2.078	0.149	1	0.149	0.245	1	8.475	**0.004**

*Porcellio dilatatus males were injected with invasive *w*VulC *Wolbachia* strain and their survival as well as their mobility was recorded on the days 60, 75, and 105 PI. A mixed effects linear model with random block effect was used to analyze mobility data. A mixed effects Cox proportional hazards model was fitted using the survival data of the lineages to estimate the effects of the treatment and the *Wolbachia* status. For both models, first a global model with all of the lineages (symbiotic dilatatus A-*w*Con, dilatatus A-*w*Dil and asymbiotic dilatatus A) was fitted, then further analysis was made to clarify the differences between symbiotic dilatatus A-*w*Con and dilatatus A-*w*Dil lineages with help of a sub-model. In bold, statistically significant values (*p* < 0.05).*

#### Mobility

**FIGURE 8:**
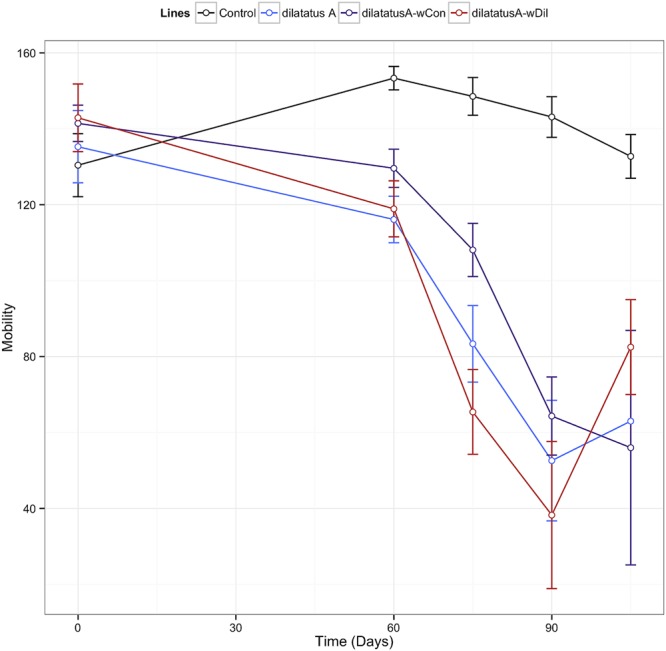
**Influence of resident *Wolbachia* on the mobility of *P. dilatatus* infected with *w*VulC.** *P. dilatatus* males were injected with pathogenic *w*VulC *Wolbachia* strain and their mobility was recorded every 15 days. The mobility test was performed measuring the time the animals move in a Petri dish during a period of 180 s. Data points indicate the mean (±SEM).

Leg tremors and seizures caused by the *w*VulC infection were observed during the course of experiment for all lines. The mobility of the animals injected with *w*VulC decreased compared to the control group, starting from the day 60 PI (**Table 3**). This decrease continued until the last days of the infection monitoring in all lines. However, the decrease of mobility due to *w*VulC was lower for symbiotic dilatatus A-*w*Con individuals compared to the other symbiotic lineage dilatatus A-*w*Dil at both days 75 and 105 PI (**Table 3**; **Figure 8**).

## Discussion

The potential mutualistic nature of *Wolbachia* as a protective symbiont was revealed few years ago by the interactions between *Wolbachia* and RNA viruses in *Drosophila* (Hedges et al., 2008; Teixeira et al., 2008). Since then, the protection conferred by the presence of *Wolbachia* has been reported against various natural enemies. However, this has mainly been observed in artificially established host-*Wolbachia* associations either when a naturally naive host was transinfected with *Wolbachia* (Kambris et al., 2009; Ye et al., 2013), or when a non-naive host (i.e., host species for which some individuals are infected by another *Wolbachia* strain) was transinfected with a new *Wolbachia* strain (Blagrove et al., 2012). The aim of our study was to find out whether a resident *Wolbachia* has a protective effect against intracellular bacterial pathogens in terrestrial isopods.

In *A. vulgare*, we showed that lines harboring the feminizing *Wolbachia w*VulC (BF*w*VulC, ZN, WX*w*) survived both *L. ivanovii* and *S. typhimurium* injections better than their asymbiotic counterparts (BF, WXa). This demonstrates that *w*VulC behaves as a protective symbiont for *A. vulgare*. Moreover, even if our analyses of the different lines coming from different populations of origins showed an influence of ‘host-background’ in the protection phenotype, the major source of variation in survival to pathogenic bacteria is clearly the absence or presence of *Wolbachia w*VulC. A prevailing effect of *Wolbachia* on *A. vulgare* physiology was already demonstrated by studying several immune parameters (Braquart-Varnier et al., 2008; Sicard et al., 2010). In 1-year-old *A. vulgare*, the same age as the animals used in the present study, the presence of *w*VulC influences both PO activities (Sicard et al., 2010) and hemocyte type proportions (i.e., increase in hyaline and semi-granular hemocyte, and decrease in granular hemocyte percentage; Chevalier et al., 2011). Besides native *w*VulC *Wolbachia* presence leads to a down regulation of some immune genes in the whole body of *A. vulgare*, including genes involved in stress response, detoxification, autophagy, AMP synthesis (including two Gram-positive AMPs: armadillidin and crustin), pathogen recognition and proteolytic cascades in ovaries (Chevalier et al., 2012), while most of these genes tend, on the contrary, to be up-regulated in the immune tissues of *Wolbachia* infected animals (Chevalier et al., 2012). The previously described immunodepressive effects of *Wolbachia* presence might be somehow compensated by the general up-regulation of immune genes specifically in the immune tissues hence causing the observed protective effect. However, the global picture of *Wolbachia* influence on *A. vulgare* immune system is not yet unraveled and the protection against bacterial pathogens demonstrated here cannot be linked to any previously demonstrated effect of *Wolbachia* presence. Besides any immune system stimulation, such protection effect of the presence of *Wolbachia* could also be the result of negative interferences between resident symbiont and invasive bacteria in diverse possible ways.

In *P. dilatatus*, the two CI-inducing *Wolbachia* strains (non-native *w*Con and native *w*Dil) both conferred protection of both females and males against pathogenic bacteria. However, each resident *Wolbachia* increased survival against different invasive pathogenic bacteria: the native *w*Dil conferred better resistance to *S. typhimurium* infection while *w*Con conferred a slightly higher resistance against *L. ivanovii*. Previous assessments of the influence of both *Wolbachia* strains on some *P. dilatatus* immune parameters (PO, hemocyte load and phagocytosis rate) indicated that the two *Wolbachia* strains influence the immune system differently: the non-native *w*Con is overall more immuno-stimulating than the native *w*Dil (Pigeault et al., 2014). *w*Con as a non-native *Wolbachia* strain of *P. dilatatus* might trigger more general immune pathways than the native *w*Dil strain does, suggesting an immune stimulation due to the presence of the foreign *Wolbachia*. However, an alternative hypothesis not implying mediation via immunity may also explain such patterns of protection: *w*Dil could interact harder with *Salmonella* and counteracts its multiplication while *w*Con would interact harder with *Listeria*. For *P. dilatatus*, we also demonstrate an effect of gender on survival: females survived better than males when challenged with pathogenic bacteria. This suggests that isopod females invest more in immunity than males, as already reported by measuring immune parameters in Pigeault et al. (2014).

Previous transinfection experiments with *P. dilatatus* as a recipient host demonstrated that *w*VulC coming from *A. vulgare* resulted in strong pathogenicity when interacting with this new host (Le Clec’h et al., 2012, 2013, 2014). In the present work, the *w*VulC strain was injected into asymbiotic (dilatatus A) but also for the first time in symbiotic animals (i.e., dilatatus A-*w*Con or dilatatus A-*w*Dil). Our results confirmed the pathogenicity of *w*VulC on *P. dilatatus* and we observed the previously reported symptoms (reduced mobility, leg tremors, seizures) suggesting a neurologic pathology (Le Clec’h et al., 2012, 2013). Nonetheless, dilatatus A-*w*Con survived longer and some symptoms such as reduced mobility were postponed compared to the other lines. The difference in survival rates following *w*VulC injections was not related to the load of resident *Wolbachia*. Therefore the reason for this difference could result from difference in the interactions between *w*VulC and the resident strain within the intracellular niche.

Our results demonstrate for the first time in any model a protection conferred by native *Wolbachia* strains against pathogenic intracellular bacteria: an important proportion (up to 70%) of the symbiotic animals survived the infection (even several weeks after infection; personal observation) while all the asymbiotic animals died. Such a strong benefit due to the presence of *Wolbachia* has only been previously reported for infection of extracellular *E. carotovora* in the naive host *A. aegypti* transinfected with non-native *Wolbachia* strains from *Drosophila* (Kambris et al., 2009; Ye et al., 2013). Other investigations on bacterial protection conferred by *Wolbachia* conducted on *D. melanogaster* naturally infected with native *Wolbachia* showed that symbiotic animals did not survive better than their asymbiotic counterparts when infected with pathogenic bacteria such as *L. ivanovii* and *S. typhimurium* as well as *Burkholderia cepacia, E. carotovora*, and *Mycobacterium marinum* (Rottschaefer and Lazzaro, 2012; Ye et al., 2013). Similarly, *Drosophila simulans* naturally infected with *Wolbachia* did not show any difference in terms of mortality compared to the flies without *Wolbachia*, when infected with *E. carotovora, Pseudomonas aeruginosa*, and *Serratia marcescens* (Wong et al., 2011). Based on these studies, it was suggested that the mechanism responsible for the protective effect against pathogenic bacteria, only observed in artificial associations between *Wolbachia* and mosquitoes, could result from an immune stimulation triggered only by non-native transinfected *Wolbachia* (Moreira et al., 2009; Bian et al., 2010; Ye et al., 2013). As such immune priming would be unlikely to be triggered in long-term associations between *Wolbachia* and their arthropod hosts, several authors concluded that a protective effect of *Wolbachia* against intracellular bacteria in natural symbiotic systems was not likely to be found (Rottschaefer and Lazzaro, 2012; Ye et al., 2013). However, in isopod models, we showed a protection against pathogenic bacteria in situations for which immune stimulation caused by the introduction of non-native *Wolbachia* in a new host cannot explain the protection phenotype. Even though in the BF*w*VulC and dilatatus-*w*Dil lines, the *Wolbachia* have been artificially introduced, the *Wolbachia* strain *w*VulC is widely distributed all over the world in *A. vulgare* populations (Cordaux et al., 2004) while *w*Dil is widely distributed in *P. dilatatus* (Grève, personal communication). Therefore, there are co-evolutionary histories between these symbiotic partners.

By measuring the concentration of pathogenic bacteria in the hemolymph of the injected animals, we investigated whether the observed protection would be due to a true resistance phenomenon which would result in reduced pathogen load, or tolerance which would result in a similar pathogen load but increased survival (Schneider and Ayres, 2008). In *S. typhimurium* infection, symbiotic BF*w*VulC (*A. vulgare*) and dilatatus A-*w*Dil (*P. dilatatus*) lines showed lower pathogen load in their hemolymph than asymbiotic BF (*A. vulgare*) and dilatatus A (*P. dilatatus*) lines respectively. Therefore, the better survival of symbiotic animals would correspond to a decrease in bacterial load in the hemolymph (i.e., higher resistance). On the other hand, we did not find any difference in *L. ivanovii* load in the hemolymph of the isopods 24 h PI (**Figure 4**) or even 48 h PI (personal observation). These results suggest that the difference in survival between lines after *Listeria* injection was not linked to difference in pathogenic bacterial loads. However, further investigations would be required to have a firm conclusion on the latter, since in our experiments *Listeria* was clearly pathogenic while we were not able to detect a proper infection (i.e., multiplication) in the hemolymph.

Maternally inherited *Wolbachia* strains induce either feminization or CI in terrestrial isopods. As these reproductive manipulations and in general *Wolbachia* infections can have severe costs on host fitness, they are considered as conflictual interactions. Indeed various *Wolbachia* strains are linked to some detrimental effects on their host’s fitness, such as *w*VulC strain in *A. vulgare* causing reduced progeny and survival (Braquart-Varnier et al., 2008; Sicard et al., 2010). Given that *Wolbachia* are quite widespread in host populations despite their apparent fitness costs, we hypothesize that the observed protection effect could compensate for the costs, and that *Wolbachia* can become a mutualist, especially if infections by environmental bacteria constitute an important threat for terrestrial isopods. Moreover in *P. dilatatus*, the native *w*Dil strain has recently been shown to increase the reproduction of its host (Pigeault et al., 2014). If antibacterial protection conferred by the same *Wolbachia* is paired with a reproductive benefit (Pigeault et al., 2014), reproductive parasitism might continue to manipulate host reproduction while being a mutualist at the same time, resulting in a so called ‘Jekyll and Hyde’ infection, where beneficial and conflictual traits co-exist together in the same symbiotic system (Zug and Hammerstein, 2014).

## Conflict of Interest Statement

The authors declare that the research was conducted in the absence of any commercial or financial relationships that could be construed as a potential conflict of interest.
